# New York Odontological Society

**Published:** 1880-03

**Authors:** Chas. E. Francis, Benj. Lord, Wm. Jarvie, W. H. Dwinelle, A. L. Northrop


### ARTICLE IX.

### *New York Odontological Society.*

The committee appointed in reference to the death of our esteemed friend, Dr. Samuel S. White, submit the following as their report:

The intelligence of the sudden and unexpected death of our friend and co-laborer, Dr. Samuel S. White, of Philadelphia, awakens within our hearts emotions of the most profound sorrow; and while bowing to the Divine wisdom which has taken him away from us, we are impelled to take the first opportunity of paying a grateful tribute to his memory, and to give expression of our appreciation of him, as a man. In so doing, our minds naturally revert to his earlier career, and thence forward to the unfortunate hour which closed a life full of promise, usefulness, honor, and success. All will bear testimony that he was a genial and cordial gentleman of high culture and noble impulse, full of kindly charities, and always alive to the interest of our profession. With us he was ever a welcome guest, cheering and encouraging us in our aspirations, and giving character and tone to our deliberations.

His relations to our profession were of a peculiar character. He was one of us from the beginning. Commencing as a student and following along through a varied career to his graduation. His interests have ever run parallel with our development and growing needs. He was then better qualified than any other man to respond to the demands of our ever advancing profession. Enterprising even to daring. He never failed to take every rational opportunity to develop and establish any prospective good to our calling. While recognizing the hand of Providence in thus removing from our midst in the very fullness of his prime and usefulness, our friend, Dr. Samuel S. White, and while deploring our own loss, we do as a Society, tender to his family and friends our heartfelt expression of sympathy and condolence.

CHAS. E. FRANCIS,
BENJ. LORD,
WM. JARVIE, JR.
W. H. DWINELLE,
A. L. NORTHROP.

